# Aptamer–Target–Gold Nanoparticle Conjugates for the Quantification of Fumonisin B1

**DOI:** 10.3390/bios11010018

**Published:** 2021-01-08

**Authors:** Vicente Antonio Mirón-Mérida, Yadira González-Espinosa, Mar Collado-González, Yun Yun Gong, Yuan Guo, Francisco M. Goycoolea

**Affiliations:** School of Food Science and Nutrition, University of Leeds, Leeds LS2 9JT, UK; y.gonzalezespinosa@leeds.ac.uk (Y.G.-E.); m.d.m.colladogonzalez@leeds.ac.uk (M.C.-G.); y.gong@leeds.ac.uk (Y.Y.G.); y.guo@leeds.ac.uk (Y.G.)

**Keywords:** Fumonsin B1, aptamers, gold nanoparticles, UV/VIS spectroscopy, asymmetric flow field-flow fractionation

## Abstract

Fumonisin B1 (FB1), a mycotoxin classified as group 2B hazard, is of high importance due to its abundance and occurrence in varied crops. Conventional methods for detection are sensitive and selective; however, they also convey disadvantages such as long assay times, expensive equipment and instrumentation, complex procedures, sample pretreatment and unfeasibility for on-site analysis. Therefore, there is a need for quick, simple and affordable quantification methods. On that note, aptamers (ssDNA) are a good alternative for designing specific and sensitive biosensing techniques. In this work, the assessment of the performance of two aptamers (40 and 96 nt) on the colorimetric quantification of FB1 was determined by conducting an aptamer–target incubation step, followed by the addition of gold nanoparticles (AuNPs) and NaCl. Although MgCl_2_ and Tris-HCl were, respectively, essential for aptamer 96 and 40 nt, the latter was not specific for FB1. Alternatively, the formation of Aptamer (96 nt)–FB1–AuNP conjugates in MgCl_2_ exhibited stabilization to NaCl-induced aggregation at increasing FB1 concentrations. The application of asymmetric flow field-flow fractionation (AF4) allowed their size separation and characterization by a multidetection system (UV-VIS, MALS and DLS online), with a reduction in the limit of detection from 0.002 µg/mL to 56 fg/mL.

## 1. Introduction

Exposure to FB1 occurs not only in African [1] and Latin-American [2] countries but also in several regions of Asia [3] and Europe [4]. For that reason, monitoring and controlling contamination of food commodities with fumonisins becomes a highly pressing matter for protecting human health worldwide. Fumosinins are a group of toxins generated by diverse fungi including *Fusarium verticillioides* [5], *Alternaria alternata* [6], *Aspergillus niger* [7], *Tolypocladium cylindrosporum*, *Tolypocladium geodes* and *Tolypocladium inflatum* [8]. Their chemical structure commonly consists of alkylamines, whose substitution of up to seven side chains allows the formation of different analogs, group B being the most common in nature [9]. From the latter, Fumonisin B1(C_34_H_59_NO_15_) has been reported to be a latent risk in several food products, such as cereals and beverages.

Classified as group 2B hazard, FB1 (Figure S1) has a possible carcinogenic effect on human health [10], whose toxicity has been related to the disruption of sphingolipid metabolism, oxidative stress and epigenetic changes [11], along with the interruption of barrier functions [12]. Nevertheless, current conventional methods for mycotoxin detection, including enzyme-linked immunosorbent assay (ELISA), high-performance liquid chromatography with fluorescence detection (HPLC-FLD) and liquid chromatography-mass spectrometry (LC-MS), are costly, time consuming, are difficult to be applied on site and require experienced users [13]. Therefore, there is a need for developing sensitive, yet quick and affordable methods for quantifying mycotoxins.

Biosensors are a suitable alternative to conventional methods by means of their general mechanism, where any target detection is carried out by a biological receptor and transduced into a signal (optical, electrochemical, mechanical, etc.). From the different biorecognition receptors (enzymes, antibodies, nuclei acids, cells, etc.), the performance of aptamers has been exceptional [14]. Aptamers are single-stranded DNA or RNA molecules with high molecular recognition toward different types of molecules, distinct binding affinities, target selectivity and high capability to discriminate slight chiral differences [15]. Their selection technique called Systematic Evolution of Ligands by Exponential enrichment (SELEX), involves incubating a DNA library with the specific target or other relevant molecules, followed by the amplification of potential binders after several selection and discrimination rounds [16]. In addition to their good performance, compared to antibodies, aptamers are easy to modify, as well as reproducible by solid-phase chemical synthesis, which represents a reduction in cost and time. To date, two aptamers specific for FB1 composed of 96 and 80 nucleotides (nt) have been selected by SELEX and used in up to 31 different biosensing approaches [15,16], through aptamer modifications, hybridization with complementary strands or reduction in the sequence length (Figure S2). Those applications have involved optical, chemiluminescence, electrochemical, surface-enhanced Raman spectroscopy (SERS), mass spectrometry (MS) and bending related signals, where the most sensitive methods were correlated with fluorescent and SERS read outs (Figure S2).

A decisive step during the design of aptamer-based biosensors is the selection of the target-aptamer incubation conditions (buffer, time and temperature). In such approaches, Tris, Tris-HCl, phosphate buffer, NaCl, CaCl_2_, KCl and MgCl_2_ are normally added during the binding stage, which normally results in a 3D conformational change of aptamers upon binding [16]. Apart from the sensitive response obtained through aptamer dehybridization from complementary sequences at specific binding sites, mycotoxin detection has been carried out by immobilization of aptamers onto the surface of different platforms such as graphene and gold nanoparticles. In this regard, gold nanoparticles (AuNPs) are suitable for developing colorimetric methods, which are still relevant due to its feasible application for on-site analysis and reduction in engineering costs. The thermodynamic instability of colloidal gold suspensions potentiates their flocculation. This aggregation, however, can be prevented when gold nanoparticles are coated with negatively charged citrate molecules, or at low ionic strength and pH above the isoelectric point of citrate. Particle aggregation in this case occurs when an aqueous dispersant medium containing the nanoparticles has a high enough ionic strength to screen their electrostatic repulsion charges caused by their citrate stabilization (e.g., salt-induced aggregation) [17]. AuNPs are commonly analyzed in terms of their absorbance by using surface plasmon resonance related to their color modification (red to blue) by aggregation with cationic compounds, changes in the suspension pH and ionic strength [18,19]. The SPR spectra of AuNPs are closely related to their particle size, which plays an important role in their absorption, scattering and excitation behavior [20]. Functionalization of AuNPs can be promoted through aptamer (ssDNA) uncoiling, whose bases are exposed to the negative surface of AuNPs, thus enabling their interaction by van der Waals forces [21].

In addition to the spectrophotometric analysis of AuNPs, particle size characterization can be achieved by more robust methods capable of probing the interaction between aptamers and AuNPs in the presence of a target molecule. For instance, asymmetric flow field-flow fractionation (AF4) is a technique that allows the separation of particles and aggregates in a size range from 1 nm to 1 µm, based on their diffusion coefficient in aqueous media. This analytical method offers several advantages, such as minimal sample alteration and efficient quantitative analysis of the physico-chemical parameters of the study materials such as their concentration and particle size by multidetection (UV/VIS, fluorescence, multiangle light scattering (MALS) and differential refractive index (dRI)) [22]. The general AF4 operation principle is based on the generation of a parabolic laminar flow profile within the separation channel transporting the particles. The action of a perpendicular flow, known as cross flow (CF) across the semipermeable membrane, drives separation according to the diffusion coefficient of particles. Some AF4 methods have been developed for studying the properties of AuNPs as either standard samples [22] or additives [23], as well as to study the target-binding relation between aptamers and their specific targeted proteins [24].

In this paper, we developed a bulk colorimetric method to examine the efficiency of two aptamers, namely, the first 96 nt sequence and a shortened version (40 nt) from the 80 nt aptamer, to quantify FB1 in different binding buffers and assessed its potential as a robust biosensing method. The general procedure involved incubating FB1 with aptamers, followed by another incubation stage with AuNPs, addition of NaCl and subsequent analysis by UV/VIS spectroscopy (λ = 400–800 nm). In addition, the characterization of the formation of Aptamer (96 nt)–FB1–AuNP conjugates was carried out by multidetection AF4, thus uncovering a promising application on the sensitive detection of this mycotoxin.

## 2. Materials and Methods

### 2.1. Materials

Fumonisin B1 (FB1, CAT FB1147), aflatoxin B1(AFB1, CAT A6636) from *Aspergillus flavus*, ochratoxin A (OTA, CAT 32937) and sodium azide (CAT 71290) were obtained from Sigma-Aldrich (St. Louis, MO, USA). Tris-HCl Buffer (UltraPure™ 1M pH 7.5, CAT 15567027) was acquired from Invitrogen™(USA). Sodium chloride (CAT BP358-1), methanol (CAT A454-1) and phosphate buffered saline (PBS, pH 7.4, CAT BP2944-100) tablets were purchased from Fisher Scientific (Loughborough, UK). Magnesium chloride (CAT J364) and potassium chloride (CAT 1.04936) were both bought from VWR (Lutterworth, UK) and BioChemica (Barcelona, Spain), respectively. Novachem surfactant 100 (C-SUR-100, lot 162167) was purchased from Postnova Analytics (Landsberg am Lech, Germany). All experiments were conducted using Mili-Q water (MQ water). Synthesized, dried and HPLC-purified aptamers specific for FB1 (Aptamer 40 nt: 5′-C GAT CTG GAT ATT ATT TTT GAT ACC CCT TTG GGG AGA CAT-3′ and Aptamer 96 nt: 5′-ATA CCA GCT TAT TCA ATT AAT CGC ATT ACC TTA TAC CAG CTT ATT CAA TTA CGT CTG CAC ATA CCA GCT TAT TCA ATT AGA TAG TAA GTG CAA TCT-3′) were purchased from Biomers.net (Germany) and diluted in sterile Milli-Q water.

### 2.2. Synthesis and Characterization of Gold Nanoparticles (AuNP)

Two stock solutions (stock 1 and 2) of gold nanoparticles (AuNP) were synthesized by citrated reduction [25]. The concentration of AuNP was determined according to the Lambert–Beer equation, Equation (1), based on a wavelength scan (200–800 nm) performed using a Specord 210 Plus Analytic Jena spectrophotometer (Jena, Germany).

(1) A=C∗ε∗L

where *A* is the absorbance, *C* the molar concentration, *ε* is the molar extinction coefficient (3.67 × 10^8^ M^−1^cm^−1^-specific for AuNP with a surface plasmon resonance (SPR) peak wavelength of 520 nm) and *L* the path length (1 cm).

Particle size distribution (nm) of AuNPs was determined in triplicate by dynamic light scattering with non-invasive back scattering (DLS-NIBS) at a measurement angle of 173° at 25 °C, in a Malvern Zetasizer NanoZS (Malvern Instruments, UK) fitted with a red laser (λ = 632.8 nm), and software in automatic mode.

### 2.3. Functionalization of Gold Nanoparticles (AuNP) with Aptamers

To perform the functionalization of AuNPs with aptamer, first, AuNPs (30 µL) were separately mixed with different concentrations of NaCl (1:1 *v*/*v*) to find their SPR peak shifting point (aggregation point). Then, different aptamer:AuNP mol ratios were mixed and incubated for 90 min at room temperature (RT~ 22 °C), from which 30 µL was mixed in a 96-well microplate with the selected NaCl molar concentration (1:1 *v*/*v*). Measurements to find the point of aptamer functionalization (until non-aggregation was observed) were conducted through a 400–800 nm scan using a Tecan Spark 10 M plate reader (Tecan, Reading, UK).

### 2.4. Assays with Aptamer 40 nt

#### 2.4.1. Effect of Tris-HCl, PBS, and Its Combination on the Binding Effect of Aptamer 40 nt

**Figure 1:**
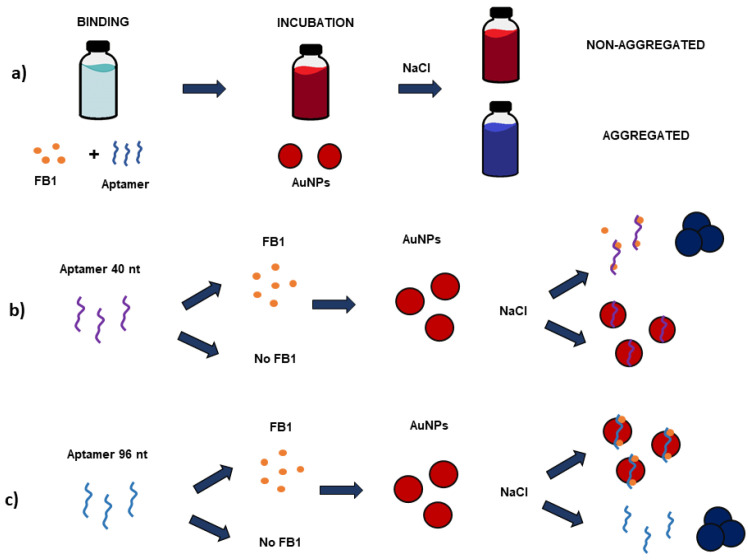
(**a**) Bulk aptasensor for the colorimetric determination of FB1with both aptamers through the binding mechanism proposed for the quantification of FB1 with (**b**) aptamer 40 nt and (**c**) aptamer 96 nt.

Three concentrations of FB1 (0, 10 and 100 µg/mL), including two high values to secure and observable effect, were dissolved in either Tris-HCl buffer 31.1 mM, PBS 12.79 mM (NaCl equivalents) or a combination of both, and were then incubated with aptamer 40 nt in a vial (calculated to a final volume of 5 µL per microplate-well), for 60 min at 37 °C. After this step, a volume of AuNPs (stock 1) necessary to reach the selected aptamer:AuNP molar ratio (117:1) was added and incubated at 37 °C for 2 h. From this solution, 30 µL was placed on a microplate well and combined with NaCl 0.4 M (1:1 v:v) and subsequently subjected to a wavelength scan (λ = 400–800 nm) on a Tecan microplate reader. The absorbance ratio at 650 and 520 nm (A_650/520_) was then calculated. The general procedure applied as a biosensing technique in this work is outlined in Figure 1a.

#### 2.4.2. Effect of Different Buffers on the Performance of Aptamer 40 nt

An increasing concentration of FB1 dissolved in different binding buffers was combined with aptamer 40 nt (final volume: 5 µL per well), for 60 min at 37 °C. The addition of AuNPs (stock 1) to achieve a molar ratio of 117:1 was conducted according to Table 1. The A_650/520_ ratio values after the addition of NaCl 0.4 M (1:1 v:v) were used to calculate the limit of detection (*LOD*) for each assay, according to Equation (2) [26].

(2) $$LOD=Blank+3\sigma_{blank}$$

where the Blank accounts for the signal exhibited by the blank (sample with no FB1), and 3σ_blank_ is three times the standard deviation of the blank. The proposed mechanism for aptamer 40 nt is displayed in Figure 1b.

#### 2.4.3. Reduction in the Aptamer: AuNP Molar Ratio

A folding step was integrated by placing a vial containing Aptamer 40 nt (dissolved in the binding buffer for this section), in a water bath at 94 °C for 5 min, followed by 15 min on ice. FB1 was also dissolved in binding buffer (Tris-HCl buffer 15 mM, NaCl 85 mM, CaCl_2_ 1mM, KCl 5mM and MgCl_2_ 2mM), and then incorporated (final volume: 5 µL per well) and incubated for 60 min at RT. Stock 1 of AuNPs was added to achieve the required aptamer:AuNPs molar ratio (47:1 to 117:1) and left in a shaking incubator (Titramax 1000, Heidolph, UK) at 300 RPM for another 60 min at RT, followed by the addition of NaCl 0.4 M (1:1 ratio v:v) and calculation of its A_650/520_ ratio and LODs.

#### 2.4.4. Specificity of Aptamer 40 nt

The specificity of aptamer 40 nt was tested on the addition of FB1, OTA or AFB1 (13.8 µM), with an aptamer:AuNPs molar ratio of 117:1 in Tris-HCl 14.06 mM with 1 h binding at 37 °C and 105 min of AuNP functionalization at RT. In addition, an aptamer:AuNPs ratio of 47:1 in buffer from Section 2.4.3 (folding included) was used for testing the specificity against OTA after 1 h binding (RT) and 1 h functionalization (RT).

### 2.5. Assays with Aptamer 96 nt

#### 2.5.1. AuNP Functionalization with Aptamer 96 nt

Aptamer 96 nt was dissolved in MgCl_2_ 1 mM and folded through a 5 min incubation in a water bath (94 °C), followed by 15 min immersion on ice. Then, different concentrations of FB1 (0.01–10 µg/mL) in MgCl_2_ 1 mM were added and incubated at 37 °C for 30 min, followed by the addition of stock 2 AuNPs (30:1 molar ratio) for 1h at RT. A wavelength scan (λ = 200–800 nm) was performed on 30 µL of the mixture combined in a 96-well microplate with NaCl 0.2 M (1:1 v:v) for calculating the A_650/520_ ratio. Additionally, the difference in absolute area was calculated in Origin Pro v. 8.6 software, between the curves of each sample and the respective blank (without FB1). The proposed mechanism for aptamer 96 nt is displayed in Figure 1c.

#### 2.5.2. Specificity of Aptamer 96 nt

The specificity of aptamer 96 nt was also tested on the addition of FB1, OTA or AFB1 (1.38 µM), by following the incubation conditions in Section 2.5.1. Such mycotoxins were selected due to their relevance and simultaneous occurrence in some food products (also related to some synergistic effects).

#### 2.5.3. Asymmetric Flow Field-Flow Fractionation (AF4)

After the functionalization of AuNPs with aptamer 96 nt at different FB1 concentrations (0.001–10 µg/mL), as indicated in Section 2.5.1, NaCl 0.2 M was added. The suspensions were subjected to AF4 conducted in an AF2000 Multiflow system from Postnova Analytics UK Ltd. (Malvern, UK). The method for the size separation of AuNPs stabilized with the aptamer–FB1 complex occurred within the channel (Postnova Z-AF4-CHA-611) having a 350 µM spacer and was performed in a 10 kDa cut-off regenerated cellulose membrane (Z-AF4-MEM-612-10KD). The temperature of the channel was controlled by a thermostat (PN4020) and set at 30 °C for all experiments. A solution of 0.05% Novachem^®^ with sodium azide (3 mM) to avoid bacterial growth in the channel was used as the carrier liquid and prepared in Milli-Q water filtered through a 0.1 µm membrane filter (VCWP Millipore). The autosampler was equipped with a 500 µL loop allowing the injection of a 100 µL sample. All the samples were measured using the following optimized AF4 method that first consisted in an injection step at a flow of 0.2 mL/min; the sample was then focused for 6 min at a rate of 1.30 mL/min with a cross-flow (CF) set at 1 mL/min. After the focusing step and a transition period of 0.2 min, the CF was decreased with an exponent decay as follows: CF was kept first constant at 1 mL/min for 0.2 min, then decreased with an exponent decay of 0.2 to 0.1 mL/min over a 40 min period and finally kept constant at 0.1 mL/min for a further 20 min. The detector flow was kept along the process constant at 0.5 mL/min to ensure detectors baseline stability. Eluted samples were finally passed and analyzed through a series of online multiple detectors: first through a dual UV/VIS detector (PN3211) set at λ = 520 and 600 nm, a refractive index detector, RI (PN3150), a 21 angle multiangle light scattering detector, MALS (PN3621) and finally through an online dynamic light scattering detector (Zetasizer Nano ZS). All recorded signals were analyzed at increasing concentrations of FB1. Specifically, the areas under UV and MALS signals were determined in Origin Pro v. 8.6 software, with normalization of the base line from each fractogram.

#### 2.5.4. Prediction of the Aptamer Folded Structure

The folded structure of nucleic acids was predicted using MFold Web Server, according to the folding conditions for aptamer 40 nt and aptamer 96 nt.

#### 2.5.5. Circular Dichroism Spectroscopy

Far-UV circular dichroism (CD) spectroscopy was conducted in a Jasco J715 spectropolarimeter with a 6-cell changer and Peltier temperature control, from λ = 200 to 340 nm. The incubation was performed as described in Section 2.5.1, at a concentration of 10 µg/L for FB1.

## 3. Results and Discussion

### 3.1. Effect of Buffer Incubation on the Quantification of FB1 with Aptamer 40 nt

The UV/VIS absorption spectra obtained for the citrate-stabilize gold nanoparticles (AuNPs) in stock 1 (6.93 nM) had a maximum peak at a wavelength of 520 nm. The aggregation of colloidal gold nanoparticles, due to charge screening, is produced by salts and cationic compounds, and can be visually observed by a change in the dispersion color from red to blue, and spectrophotometrically confirmed by a peak shift from the absorbance from λ = 520 to ~650 nm. As denoted in Figure S3a, the properties for stock 1 (Size = 18.49 ± 0.4 nm, Pdl = 0.199 ± 0.017) indicated an aggregation point after the addition of NaCl 0.4 M in a 1:1 v:v ratio (Figure S3b). Hence, this point served as the main reference for functionalization with aptamer 40 nt.

Before any incubation with aptamers, the effect of different buffers was tested on both AuNP stock solutions. A key finding was uncovering the effect that Tris-HCl buffer 50 mM (pH 7.5) exerted on the resulting aggregation of both AuNP stocks (1 and 2). Therefore, we conducted a more detailed study to assess the effect of Tris-HCl and PBS ionic strength. This study revealed that 33 mM was the maximum buffer concentration capable of inducing particle aggregation. On the other hand, the concentration of NaCl in PBS to reach the aggregation point was established at 0.4 M (data not shown). In addition, the possible aggregation effects from such buffers were diminished by an initial aptamer–target incubation, and the later addition of AuNPs. Unlike the approach using AuNPs reported here, binding buffer formulations including concentrations as high as 20 mM CaCl_2_, 20 mM MgCl_2_ and 120 Mm NaCl have been documented with silica spheres [21,27] and fluorescence detection methods [28].

**Figure 2:**
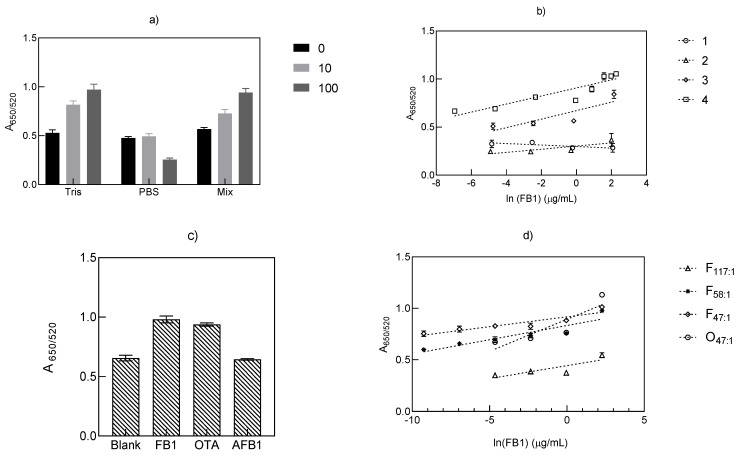
Effect of the incubation of aptamer 40 nt with FB1 on the A_650/520_ ratio in (**a**) Tris-HCl buffer 31.1 mM, PBS 12.79 mM (NaCl equivalence) and a combination of both (Mix) to a final concentration of 31.1 (Tris-HCl) and 12.79 mM (PBS in NaCl equivalence), under the same binding conditions (*n* = 6), and (**b**) different buffers at the conditions outlined in Table 1 (the numbers correspond to each assay, *n* = 3). (**c**) Specificity of assay 4 incubated with other mycotoxins (13.8 µM, *n* = 4) and (**d**) the effect of the reduction in the aptamer: AuNP molar ratio (as shown in the legend subscripts) in the incubation with FB1 (F) and OTA (O)(*n* = 4).

The study of the effect of different buffers was driven by the variety of buffers applied for aptamer-based detection of FB1 [29]. Therefore, all the tested buffer conditions in this work, were selected based on their previously reported inclusion during the binding step of several aptasensors for FB1. As shown in Figure 2a, the effect of incubation in Tris-HCl buffer (pH 7.5, 31.1 mM), PBS (pH 7.4, 12.79 mM) and its combination on the binding effect of aptamer 40 nt toward FB1 was assessed. It was observed that under the same binding conditions, Tris-HCl buffer and a Tris-HCl/PBS mixture provided optimal performance at an increasing concentration of FB1 (0–100 µM). The trend of such increments was similar between the incubation with Tris-HCl and that in the mixed buffer. In contrast, PBS resulted in an opposite effect on the A_650/520_ value. A non-significant difference was found between Tris-HCl and mixed buffer (*p* = 0.33) at the highest toxin concentration (10 µM), which denoted their similar effect on FB1 binding. Yet, as a more linear trend can be observed when employing the mixture of both buffers (Figure 2a), this was selected for exploring a wider range of mycotoxin concentrations (0.0086–8.6 µg/mL). As suggested by the linear curves shown in Figure 2b, the incubation of aptamer 40 nt with increasing FB1 concentrations, in the presence of Tris-HCl and its combination with PBS, decreased the number of available aptamer strands due to aptamer binding, so that fewer AuNPs were functionalized and thus they were not protected from NaCl-induced aggregation corresponding to the visible appearance of a blue color (Figure 1b) (Figure S4a), and an increment on the A_650/520_ value.

The result of incubating aptamer 40 nt and FB1 in the presence of four different buffers is displayed in Figure 2b. As noted, the incubation with MgCl_2_ (Assay 1) and its combination with NaCl (Assay 2) were unfavorable for the quantification of FB1, which was confirmed by the determination coefficients (r^2^) and the high LODs in Table 1. In accordance with Figure 2a, Tris-HCl and its mixture with PBS (Assays 3 and 4) afforded greater r^2^ and lower LODs, from which the sole application of Tris-HCl resulted in an enhancement effect on the method sensitivity, which resulted in an overall greater performance by means of the NH_3_^+^ group in Tris-HCl, compared to the ions (Cl^−^, Na^+^, K^+^) from PBS. Despite such confirmed effect, incubation under the presence of Tris-HCl buffer with further AuNP functionalization (Assay 4) was not specific for FB1, as corroborated in Figure 2c, where the addition of the same concentration of FB1, OTA and AFB1 did not show significant differences between the signals for OTA and FB1 (*p* = 0.065). In both cases, the aptamer–mycotoxin incubation step resulted in less unbound aptamer strands at increasing target concentrations, which diminished the number of functionalized AuNPs, hence more particle aggregation was observed. Aptamer 40 nt is a shortened version of an 80 nt sequence (Kd = 62 nM), selected in 100 mM NaCl, 20 mM Tris-HCl, 2 mm MgCl_2_, 5 mM KCl, 1mM CaCl_2_ and 0.02% Tween 20 [16]. The application of shorter sequences corresponds to an attempt to reduce synthesis costs, while increasing its affinity by selecting specific binding regions [16,30].

To apply the favorable effects of Tris-HCl buffer and reduce its lack of specificity, more cationic compounds, such as NaCl, CaCl_2_, KCl and MgCl_2_, were included, along with a reduction in the aptamer:AuNP ratio (117:1 to 47:1), and examined the improvement of the LODs as indicated in Table 2. The inclusion of counterions such as Na^+^ is intended in this case to reduce the repulsion between negative charges from the DNA backbone; calcium promotes the formation of coordination complexes with carboxyl groups present in FB1, whereas the use of monovalent ions (K^+^) is commonly applied to steady guanine tetrads [15]. Even when this strategy enhanced the sensitivity in terms of the increment of A_650/520_, from 0.54 to 0.0007 µg/mL, while simultaneously increasing the r^2^ values of the curves from Figure 2d, the incubation with OTA at a molar ratio of 47:1 (aptamer:AuNP) was highly correlated (R = 0.998, *p* = 0.002) to the values obtained through the determination of FB1 at a 58:1 molar ratio, as observed in the overlapping curves (F58:1 and O47:1) from Figure 2d.

Likewise, the equation parameters and LOD reported for OTA (0.06 µg/mL) revealed the absence of specificity from aptamer 40 nt to FB1, under the selected conditions and the biosensing technique presented in this section. The latter was opposite to the specificity reported for aptamer 40 nt through an electrochemical approach in the presence of OTA and thrombin incubated in PBS and Tris buffers [31]. Although Tris-HCl was confirmed as an ideal buffer for aptamer 40 nt, the lack of specificity could explain the existence of only two aptamer 40 nt-based methods since their disclosure in 2017 [28], and the role of the incubation conditions and sensing platform on the variable specificity of a certain sequence. In keeping with this argument, a recent study based on in silico docking assays evaluated the affinity of the 80 nt aptamer, from which aptamer 40 nt was obtained. Unlike the high affinity to free FB1, no binding was observed when FB1 was immobilized in magnetic beads [32].

Such unspecific binding can also be addressed in terms of buffer pH (7.4–7.5) in which FB1 appears as zwitterion due to the pKa values of its trycarballylic acid (3.49, 4.56, 5.83) and the amine group (pKa > 9) resulting in non-specific electrostatic interactions with aptamer 40 nt, similar to the adsorption on several materials [33], which was also observed through the role of the ionic forms of OTA from its carboxyl (pKa = 4.3–4.4) and phenolic hydroxyl groups (pKa = 7–7.3) [34]. Based on these results, we confirmed that the specificity and good performance of aptamers depends on the binding buffer and binding conditions, along with the selected sensing mechanism. Although Tris-HCl combined with PBS buffer indicated a good sensing capability from aptamer 40 nt, its lack of specificity was revealed under the presence of OTA.

### 3.2. Quantification of FB1 with Aptamer 96 nt

Stock 2 of AuNPs also had a maximum peak at a λ = 520 nm; nevertheless, the aggregation profile induced by NaCl was described by a slight reduction in the absorbance at λ = 520 nm and a pronounced peak increment from λ = 550 to 650 nm (Figure S5b). As previously mentioned, the characteristics of AuNPs are relevant to describe and understand their resulting signals. Hence, the properties of stock 2 (Size = 21.65 ± 0.22 nm, Pdl = 0.087 ± 0.016) are displayed in Figure S5a,b, where NaCl 0.2 M 1:1 ratio (v:v) indicated the main constrain for functionalization with aptamer 96 nt at an aptamer:AuNP molar ratio of 30:1 (Figure S5c). In this regard, when compared to stock 1, the application of a lower concentration of NaCl on AuNPs functionalized with aptamer 96 nt corresponds to the lower concentration of stock 2 (2.21 nM) when compared to stock 1 (6.93 nM).

From all the buffers previously tested for aptamer 40 nt, only MgCl_2_ exhibited a positive performance for the proposed bulk technique with aptamer 96 nt; therefore, it was selected for detecting FB1. The addition of 5 mM MgCl_2_ showed aggregation of stock 2 AuNPs by a colorimetric shift from red (stable AuNPs) to purple (i.e., an indicative of a certain degree of aggregation). A reduced concentration of MgCl_2_ (1 mM) was, therefore, used to diminish those negative effects. Adding Mg^2+^ ions in binding buffers contributes to their blocking effect toward the repulsion of the negative charges from the DNA backbone, which allows a more compact folding [15]. Unlike other aptasensors, it seemed that the incubation of aptamer 96 nt with FB1 in MgCl_2_ did not lead to a conformational change in its structure, thus resulting in the formation of an aptamer–FB1–AuNP complex (Figure 1c), stable against NaCl-induced aggregation at increasing concentrations of FB1 (Figures S5d and S6d). The formation of an aptamer–target–AuNP conjugate was also observed for the determination of serotonin with AuNPs and aptamers dissolved in 2 mM MgCl_2_ (pH 7.4), where a serotonin–aptamer complex was capable of protecting AuNPs from salt-induced aggregation, especially at high serotonin concentrations [35].

**Figure 3:**
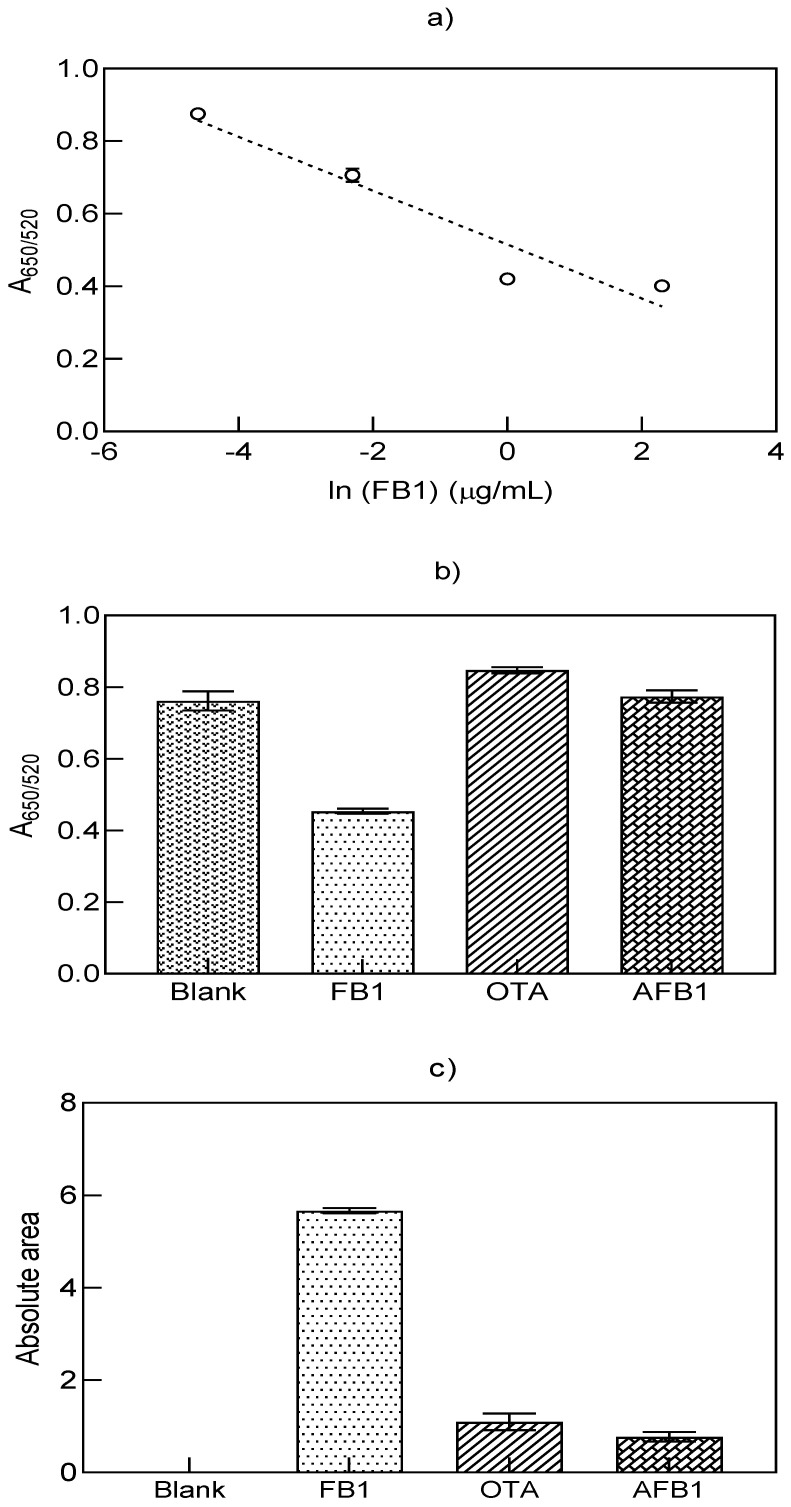
(**a**) Standard curve for the quantification of FB1 with aptamer 96 nt in MgCl_2_ through the analysis of the A_650/520_ ratio. Specificity test under the presence of FB1, AFB1 and OTA at a concentration of 1.38 µM for the (**b**) A_650/520_ ratio and the (**c**) absolute area values (*n* = 3).

As shown in Table 3, a reduction in the LOD value was achieved with aptamer 96 nt, where the value for the A_650/520_ ratio (0.003 µg/mL), calculated from the curve in Figure 3a, was lowered when the absolute area was analyzed instead (0.002 µg/mL). To increase the sensitivity of the analysis as well as minimize reproducibility issues, calculation of the absolute area was conducted. The estimation of the differential area under the curve between the blank and its corresponding standard curve points improved the linearity and slope. This mathematical comparison, where each experiment had a reference point (blank), could reduce the variability of the results when changing the stock solutions, using AuNPs with varying shapes and particle sizes, or when observing stock aggregation upon storage. Therefore, before its subtraction, the spectrum of the corresponding blank must be acquired during each batch run. Contrary to the issues observed for aptamer 40 nt, the proposed method evinced high specificity from aptamer 96 nt toward FB1 as displayed in Figure 3b,c for the A_650/520_ ratio and absolute area, respectively. In both cases, AFB1 and OTA had similar signals to the blank values, where FB1 displayed a distinct result. In different biosensing approaches, aptamer 96 nt has been confirmed as specific to FB1 in the presence of almost 19 compounds, which justifies its use in 24 biosensors [29]. From these results, we observed the positive effect of MgCl_2_ for the biorecognition of aptamer 96 nt to FB1, through the formation of an aptamer 96 nt–FB1–AuNPs conjugate. Such mechanism was specific to FB1, with a protective effect to salt-induced aggregation at increasing target concentrations.

### 3.3. Asymmetric Flow Field-Flow Fractionation (AF4) of the Aptamer 96 nt–FB1–AuNP Conjugates

**Figure 4:**
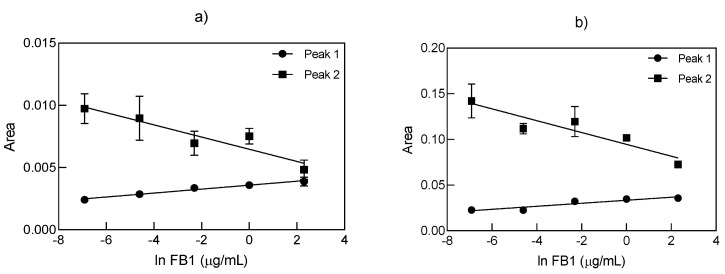

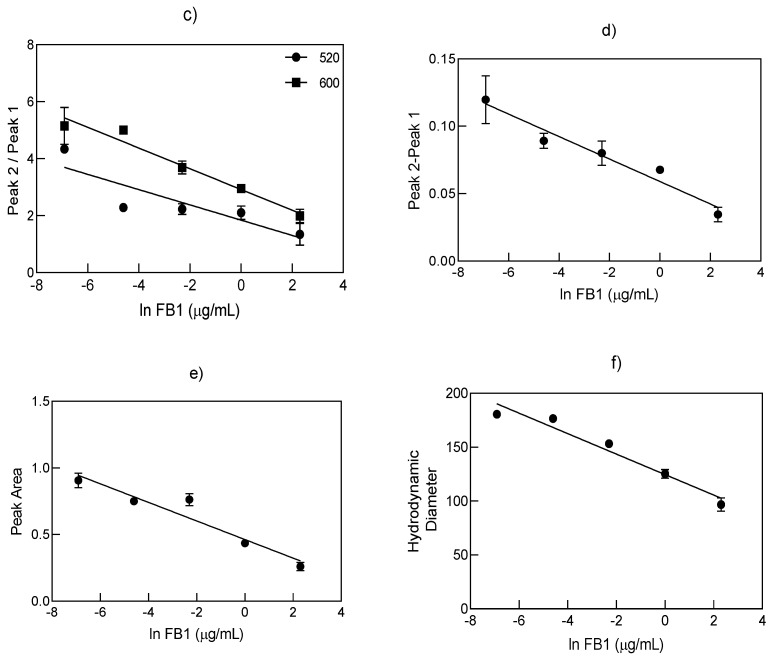
Standard curves for the quantification of FB1 with aptamer 96 nt through the analysis of the AF4 fractograms from the UV-Vis peak areas at (**a**) 520 nm, (**b**) 600 nm, (**c**) peak ratio between Peak 2 (larger particles) and Peak 1 (smaller particles), (**d**) peak area differences at 600 nm, (**e**) MALS peak area at 28° and (**f**) hydrodynamic diameter determined by DLS for the Aptamer (96 nt)–FB1–AuNPs conjugates in NaCl 0.2 M (*n* = 3).

The characterization of different aptamer 96 nt–FB1–AuNP conjugates after the addition of NaCl 0.2 M was further examined by multidetection AF4, to confirm the combined role of the aptamer 96 nt–FB1 complex in the functionalization and protection of AuNPs against NaCl-driven aggregation at different target concentration. As a first step, it was determined both visually and spectrophotometrically that the UV/VIS signal at λ = 520 and 600 nm after AF4 separation showed higher peaks at lower FB1 contents (results not shown). AF4 relies on the separation of particles of varying size depending on their hydrodynamic and diffusion properties, where the initial elution corresponded to small stabilized particles (Peak 1), whereas larger particles (Peak 2) eluted after, before passing through the detectors as shown in representative fractograms (Figures S6a and S6b). According to the AF4 principle, longer elution times correspond to particles of larger sizes, which in many cases could also be diagnostic of aggregation. Compared to the spectrophotometric analysis, AF4 contributed to the refinement of the detected signals, with a much greater resolution at lower target concentrations. Even when two peaks were detected at λ = 520 and 600 nm, the peak corresponding to the highest particle sizes (Peak 2) had a more favored trend for its potential application in biosensing techniques, as displayed in Figure 4a,b. The selection of this peak was consistent with the lower LODs achieved when analyzing the values of Peak 2 at both wavelengths (Table 3). The UV/VIS signal at λ = 600 nm was mainly ascribed to the aggregation profile of AuNPs; for that reason, the peaks between 40 and 60 min were larger and more noticeable among different concentrations of FB1 (Figure S6b).

The reported analytical method demonstrated the presence of stabilized and aggregated particles in the same suspension, rather than their complete aggregation or stabilization through aptamer FB1 functionalization. Such profile was regulated by the concentration of FB1 where fewer aggregated particles were detected at higher target concentrations (Figure S6b). The presence of different particle sizes could also be attributed to the native heterogeneity of the selected stock, which is a common issue in AuNPs, derived during their manufacturing/synthesis process, and previously confirmed by AF4 as an arrangement of particles with different size, shape and zeta potential [36].

To achieve a better separation resolution of different particle populations, a regenerated cellulose membrane was reported due to its high recovery of AuNPs [22], associated with its charge, which depends on the pH value of the carrier solvent. Here, as the carrier solvent has a pH = 9.5, the regenerated cellulose membrane bears a net negative charge (zeta potential < ~−20 mV) [37], which decreases the interaction between the membrane and AuNPs, thus favoring their elution. In addition, the application of a combination of ionic and non-ionic surfactants, such as Novachem^®^ 0.05%, in the carrier solvent was considered for preventing particle aggregation and accumulation on the cellulose membrane, as reported for other surfactants, which also improved the retention profiles [38]. Similarly, surfactants also decrease particle loss, null peaks, particle separation and recovery loss [22].

Even though the retention time during AF4 fractionation can be correlated with particle size, and the MALS signal detection can be used for the determination of the radius of gyration [38,39], in our study, we focused on the analysis of the peak areas of the different signals. Therefore, the effect of FB1 on the functionalization of AuNPs with aptamers was studied as a whole system, regardless of the differences in the absolute particle size. Despite the initial characterization purpose for the application of AF4 on the conjugates at varying FB1 concentrations, the low LODs achieved, especially after analyzing the peak areas at 600 nm (56 fg/mL), unveiled its promising usage as a biosensing technique.

As highlighted in the equations and LODs (Table 3) from Figure 4c,d, neither the Peak 2/Peak 1 ratio for both wavelengths (7 and 0.6 ng/mL, respectively), nor the subtraction of Peak 1 from Peak 2 at 600 nm (1.6 pg/mL), were as sensitive as the sole analysis of Peak 2 at 600 nm. Similarly, the parameters quantified from the MALS signal at 28° (LOD = 0.16 pg/mL) and the hydrodynamic diameter obtained by DLS (LOD = 0.96 ng/mL), plotted in Figure 4e,f, respectively, resulted in lower sensitivity compared to the values determined from UV signals at 600 nm (Table 3). Although the MALS signal at 90° has been analyzed for AuNPs [38,39], in this work, more distinctive peaks between samples were found at 28°, which allowed the examination of various degrees of aggregation with a better signal-to-noise ratio. Such MALS signal (28°) was adequate for representing the effects of an increasing concentration of FB1 on the same system by analysis of its peak area, which also demonstrated that smaller angles from MALS are more sensitive to larger particles. Interestingly, the hydrodynamic diameter complied with the UV/VIS fractograms and spectrophotometric analysis, where greater hydrodynamic diameters corresponded to the presence of aggregated particles at lower FB1 contents. To the best of our knowledge, AF4 has not been yet used for the quantification of mycotoxins. One study applied this technique for quantifying the molecular weight of an aptamer–streptavidin–immunoglobulin G (IgG) complex in a biosensing technique for OTA [40]. In summary, the analysis of the aptamer 96 nt–FB1–AuNPs conjugates by AF4 revealed the presence of varied degrees of aggregated and non-aggregated particles at different target concentrations. From all the detected signals, the analysis of the UV/VIS peak area at 600 nm was the most suitable for portraying such variability, with a promising scope for the application of AF4 as a new biosensing technique in the fg/mL range.

### 3.4. Interaction of the Conjugate Elements (Aptamer 96 nt–FB1–AuNPs)

**Figure 5:**
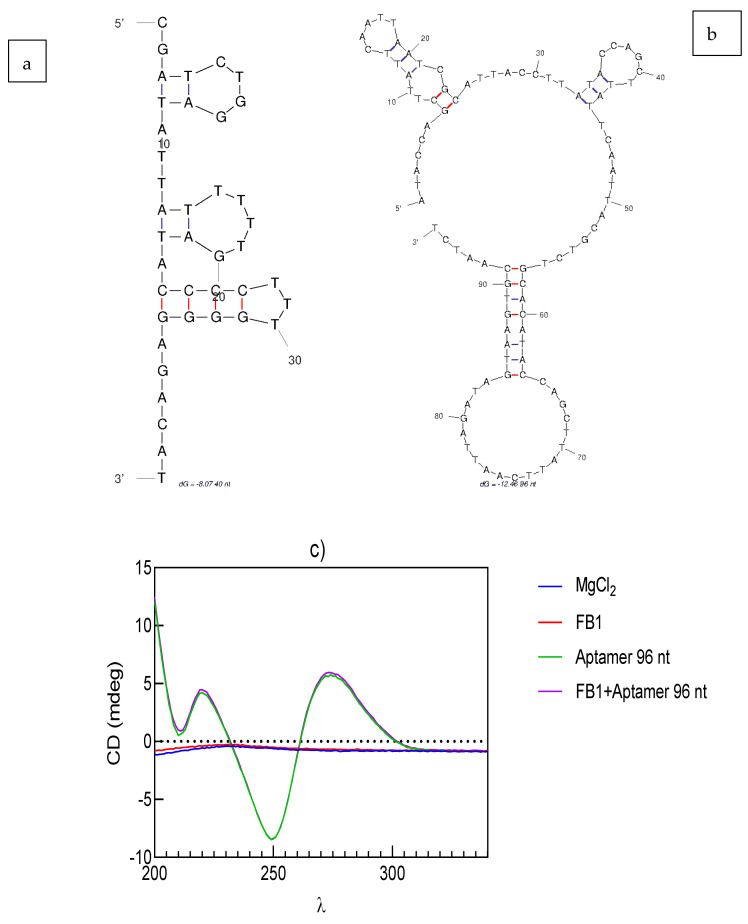
Predicted folded structures of (**a**) aptamer 40 nt (dG = −8.07), (**b**) aptamer 96 nt (dG = −12.46), and (**c**) circular dichroism spectrum of aptamer 96 nt in the absence and presence of 10 µg/L FB1 incubated in MgCl_2_.

The biosensing approach in this work did not require any complementary strand or aptamer modification (label), especially when considering that the latter might decrease its binding affinity. Here, the selection of the incubation buffer is also a relevant step for the success of the aptamer-target binding stage [41]. As shown in Figure 5a, when compared to aptamer 40 nt (dG = −6.64), the structure of aptamer 96 nt (dG = −12.43) has elongated hairpins and loops, due to its longer sequence.

A study on the binding affinity of minimers from aptamer 96 nt (Kd= 100 nM) reported that the 16 nt sequence next to the 3′ primer binding region (AGATAGTAAGTGCAATCT-3′) is related to target binding. However, the regions following the 5′ end (5′-ATACCAGCTTATTCAATT) are also necessary for the overall binding efficiencies, which correspond to the favorable dissociation constant of the sequence without both primer binding regions (60 nt, Kd = 195 nM) [30]. Nevertheless, binding assays for this minimer denoted low or null binding towards FB1 [32], which explains the majority of applications with the complete 96 nt sequence. In addition, the amine group in FB1 has been used in immobilization approaches during SELEX, motivated by the immunodominant epitope region in FB1 (close to the concurrence of tricarballylic acid to C-11 and C-20), which is distant from such functional group [16]. Even when the presence of multiple free dihedrals within the structure of FB1 requires more configurational space during binding, high binding propensity has been reported between FB1 and the backbone of different FB1 aptamers, including aptamer 96 nt [32].

Based on this, the formation of aptamer 96 nt–FB1–AuNPs conjugates first occurred by incubation of the binding region and backbone in aptamer 96 nt with its epitope zone in FB1, where a conformational change was unlikely to have occurred due to the length of the aptamer and the role of the negative backbone charges from both primer binding regions, which could have generated steric hindrance and simultaneous binding in some regions. Therefore, the conjugation was followed by immobilization of the aptamer 96 nt–FB1 complex on AuNPs by means of the NH_2_ group in FB1, which was previously evaluated on the prevented aggregation from binding buffers through the sole incubation with the target molecule, but was weak against salt-induced aggregation (Figure S4b). We thus suggest that the mechanism was completed by the formation of an aptamer 96 nt–FB1 layer by non-covalent attachment of the primer regions to the surface of AuNPs. This proposal is consistent with the increased density of aptamer 96 nt–FB1 complexes on AuNPs, and the prevented aggregation upon the addition of salt.

Circular dichroism (CD) spectra of MgCl_2_ and FB1 were very close to the solvent baseline (Figure 5c). In turn, the CD spectra for the aptamer 96 nt showed two trough bands at λ = 205 and 250 nm and a positive peak band at 280 nm region before and after binding with FB1. The CD spectrum was close to previous analysis on this sequence, which denoted helicity of its parallel arrangement [42]. The same negative (250 nm) and positive (280 nm) bands were previously described as an indicator of an A-form hairpin duplex structure, where the flat zone from 265 to 285 nm was attributed to base pair formation between A-T and G-C [16,43]. However, the 3-D representation of the most stable docked pose in aptamer 96 nt was indicated as a B-form duplex structure, as A-forms are mostly favored in RNA [32]. The peak similarity from both spectra also confirmed the absence of a conformational change upon binding. Depending on the selected immobilization and biosensing method, when a long-length aptamer is used, the FB1-modulated conformational change might not be observed, as only some regions of the aptamer display affinity. Such absence of conformational change was also confirmed by gel electrophoresis, as indicated in Figure S7, where a slight increase of 2.5% was calculated for the band intensities of aptamer 96 nt incubated with the maximum FB1 concentration (10.02 µg/mL) compared to the bands for aptamer 96 nt in MgCl_2_ 1mM. The aforementioned was opposite to the trend observed in other reports, when an increasing target concentration enhanced the band intensities from the aptamer–target complex due to conformational change [44]. To conclude, based on the CD and gel electrophoresis results, it was understood that no conformational change was found upon target binding, which also corresponded to the long structure of aptamer 96 nt, predicted in Mfold.

As a first attempt toward the subsequent full validation of the developed method, we evaluated the matrix-matched quantification of FB1 in various liquid and solid food samples. For instance, the incubation of aptamers with its target in either buffer or a corn extract showed color differences among both samples, after the addition of AuNPs (Figure S8a). Likewise, the analysis in spiked vodka displayed different aggregation spectra to those from FB1 in binding buffer (MgCl_2_ 1mM), as shown in Figure S8b,c. While spirits can be directly measured and injected into the AF4 system, a pretreatment step is required for the analysis of more complex samples such as corn extracts. As plotted in Figure S2, the LOD, for the specific quantification of FB1 by the reported aptamer–target–AuNP complex (A_650/520_ absolute area), is comparable to that for some fluorescent and electrochemical aptasensors specific for this mycotoxin. Yet, the application of AF4 is a promising technique for the analysis of FB1, which regardless of the increased assay time resulted in a low LOD value comparable with the most sensitive aptamer-based sensors reported for this purpose. The novelty of this approach lies in the integration of the unmodified 96 nt ssDNA sequence, without the inclusion of other complementary strands, supports or DNA modification (SH, NH_2_, biotin, FAM, Cy5.5). Additionally, to the best of our knowledge, this is the first report on the use of AF4 for evaluating the performance of an aptamer on the formation of a conjugate enhanced by the presence of its target molecule, thus revealing to be a highly sensitive and specific method.

## 4. Conclusions

This work presents a comparison on the performances of two aptamers for the colorimetric quantification of FB1. The results indicated that, along with the aptamer sequence, the selected buffer and incubation conditions play an important role in the final sensitivity and specificity of each assay. In this regard, incubation with Tris-HCl and MgCl_2_ was suitable for the 40 and 96-mer aptamers, respectively. Contrary to previous reports [31], the assay with a short length aptamer (40 nt) was not specific for FB1, as similar results were observed through the incubation with OTA. A different mechanism has been proposed for the long aptamer (96 nt), previously reported for several aptamer-based approaches. In this case, an aptamer–FB1–AuNP conjugate was formulated in the presence of MgCl_2_ 1 mM, showing stability to salt-aggregation at an increasing concentration of FB1(0.001–10 µg/mL). Unlike other aptasensors, the 96 nt aptamer offered a simplified approach as an unmodified ssDNA sequence was applied without the need of end modifications or complementary strands. Analysis of the spectrophotometric signals resulted in LODs similar to other sensitive techniques; however, the exploration of the aggregation profile by AF4 with multidetection (UV/VIS, MALS, DLS) derived in a promising sensing technique with sensitivity in the fg/mL level. The characterization of the complex formation revealed the absence of DNA conformational change upon binding, yet this new mechanism might be suitable for the direct analysis of different food matrices, where there is scope for exploring other targets, such as emerging mycotoxins. To our knowledge, this is the first aptasensing technique for FB1 applying the 96 nt aptamer sequence without any end modification, label or complementary strand. Likewise, there is no evidence for the use of AF4 in the exploration of aptamer–target–AuNPs interactions.

Further validation and standardization steps are still required for the commercial application and possible scaling to paper-based techniques, which might enhance the opportunities for on-site quantifications, while decreasing the total manufacturing cost. Nevertheless, this work established a new mechanism for detecting FB1 with a 96 nt aptamer in bulk, while at the same time presents for the first time the use of a more robust method, as it is AF4, resulting in LODs with strong advantages over more complex designs.

## Figures and Tables

**Table 1:** Different binding conditions for aptamer-based quantification of FB1 (aptamer 40 nt) ^1^.

Assay	Binding Buffer	Incubation with AuNP Time/Temperature (min/°C)	Equation A_650_/A_520_ =	r^2^	Range of Tested Concentrations (µg/mL)	Limit of Detection(LOD) (µg/mL)
1	MgCl_2_ 0.19 mM	120/37	−0.008 ln [X] + 0.2977	0.6542	0.008–8.0	4.16
2	NaCl 0.06M + MgCl_2_ 0.1mM	120/37	0.0164 ln [X] + 0.3025	0.6795	0.0074–7.4	0.35
3	PBS (eq. 12 mM NaCl) + Tris-HCl buffer 17 mM	120/37	0.0442 ln [X] + 0.6709	0.737	0.0086–8.6	0.11
4	Tris HCl buffer 14.06 mM	105/20	0.0419 ln [X] + 0.9072	0.8311	0.0096–9.66	0.03

^1^ X = FB1concentration in µg/mL. Assay conditions: Aptamer:AuNP (mol:mol):117:1; FB1–aptamer incubation: 60 min (37 °C).

**Table 2:** Equations and LODs for the aptamer-based quantification of FB1 with aptamer 40 nt at different molar ratios.

Assay ^1^	Equation ^2^ A_650/520_ =	r^2^	Range of Tested Concentrations (µg/mL)	LOD (µg/mL)
F_117:1_	0.0248 ln[X] + 0.4434	0.695	0.0096–9.66	0.54
F_58:1_	0.0276 ln [X] + 0.8332	0.833	0.00096–9.66	0.001
F_47:1_	0.0192 ln [X] + 0.9169	0.84	0.000096–9.66	0.0007
O_47:1_	0.0624 ln [X] + 0.8937	0.76	0.0096–9.66	0.06

^1^ F: Assays with FB1; O: Assays with Ochratoxin A; Numbers as subscript indicate the aptamer: AuNP molar ratio; ^2^ X = FB1concentration in µg/mL. Note: Buffer: Tris-HCl buffer 15 mM + NaCl 85 mM + CaCl_2_ 1 mM + 5 mM KCl + MgCl_2_ 2 mM; FB1 aptamer incubation: 60 min (RT); incubation with AuNP: 60 min RT.

**Table 3:** Equations and LOD for aptamer-based quantification of FB1 with aptamer 96 nt through different signals ^1^.

Signal		Equation	r^2^	Range of Tested Concentrations (µg/mL)	LOD (µg/mL)
A_650/520_		A_650/520_ = −0.074 ln [X] + 0.5153	0.9179	0.01–10	0.003
Absolute Area		AA = 1.34 ln [X] + 8.298	0.9243	0.01–10	0.002
Peak Area 520 nm	Peak 1	Area = 0.0002 ln [X] + 0.0036	0.9763	0.001–10	1.68
	Peak 2	Area = − 0.0005 ln [X] + 0.0065	0.8735	0.001–10	0.0001
Peak Area 600 nm	Peak 1	Area = 0.0017 ln [X] + 0.0334	0.872	0.001–10	7.83
	Peak 2	Area= − 0.006 ln [X] + 0.0946	0.858	0.001–10	0.000000056
Peak 2 Area /Peak 1 Area	520 nm	P1/P2 = − 0.268 ln [X] + 1.8427	0.7639	0.001–10	0.007
	600 nm	P1/P2 = − 0.364 ln [X] + 2.9186	0.9637	0.001–10	0.0006
Peak 2 Area–Peak 1 Area	600 nm	P–P2 = − 0.008 ln [X] + 0.059	0.9525	0.001–10	0.0000016
28^o^		Area = − 0.07 ln [X] + 0.4632	0.9128	0.001–10	0.00000016
Diameter		D = − 9.498 ln [X] + 124.61	0.9514	0.001–10	0.000959

^1^ X = FB1concentration in µg/mL. Buffer: MgCl_2_ 1mM; FB1 aptamer 96 nt incubation: 30 min (37 °C); incubation with AuNP: 60 min (RT); AuNP:Aptamer molar ratio 30:1.

## Data Availability

The data presented in this study are available on request from the corresponding author. The data are not publicly available due to its classification as intellectual property by the University of Leeds.

## Supplementary Materials

The following is available online at https://www.mdpi.com/2079-6374/11/1/18/s1.
