# Supplementary material for: Aptamer–Target–Gold Nanoparticle Conjugates for the Quantification of Fumonisin B1
Source: Biosensors (Basel). 2021 Jan 8;11(1):18. doi: 10.3390/bios11010018 (PMC7827823; doi:10.3390/bios11010018)
Supplement: Supplementary file 1 [file biosensors-11-00018-s001.pdf]

*Supplementary Information for:*

# Aptamer-target-gold nanoparticle conjugates for the quantification of fumonisin B1

Vicente Antonio Mirón-Mérida[1,*], Yadira Gonzalez-Espinosa[1], Mar Collado-González[1], YunYun Gong[1], Yuan Guo[1], Francisco M. Goycoolea[1,*]

[1]School of Food Science and Nutrition, University of Leeds, Leeds LS2 9JT, UK; y.gonzalezespinosa@leeds,.ac.uk (Y.G.E.); m.d.m.colladogonzalez@leeds.ac.uk (M.C.G.); y.gong@leeds.ac.uk (Y.Y.G.); y.guo@leeds.ac.uk (Y.G.)

* Correspondence: fsvamm@leeds.ac.uk (V.A.M.M); f.m.goycoolea@leeeds.ac.uk (F.M.G)

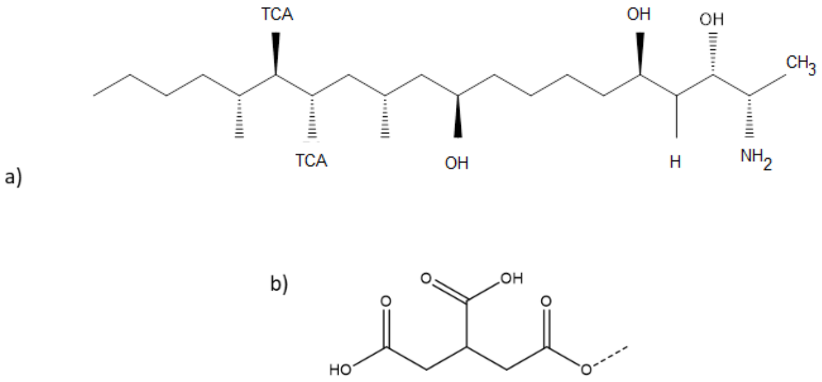


**Figure S1.** Structure representation of (a)FB1 and (b)tricarballylic acid (TCA).

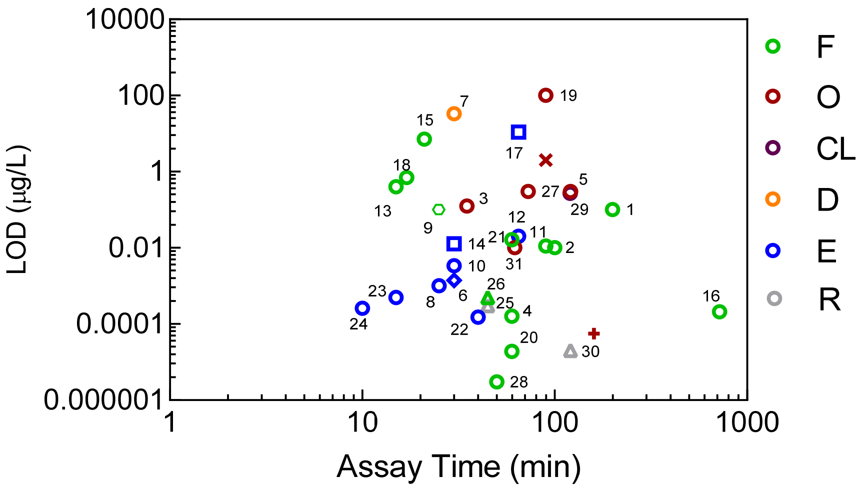


**Figure S2.** Comparison of the assays from this work with aptamer 96 nt for the analysis of the $A_{650/520}$ ratio (**X**) and the AF4 peak 2 area at 600 nm (+) with other aptamer-based biosensors with fluorescent (green), optical (red), chemiluminescent (purple), deflection (yellow), electrochemical (blue) and Raman (grey) determinations with a 96 nt (circle), 80 nt (rhombus), 60 nt (hexagon), 40 nt (square) and not specified (triangle) sequence. Each labelled number represents a reference listed at the end of the supplementary materials.

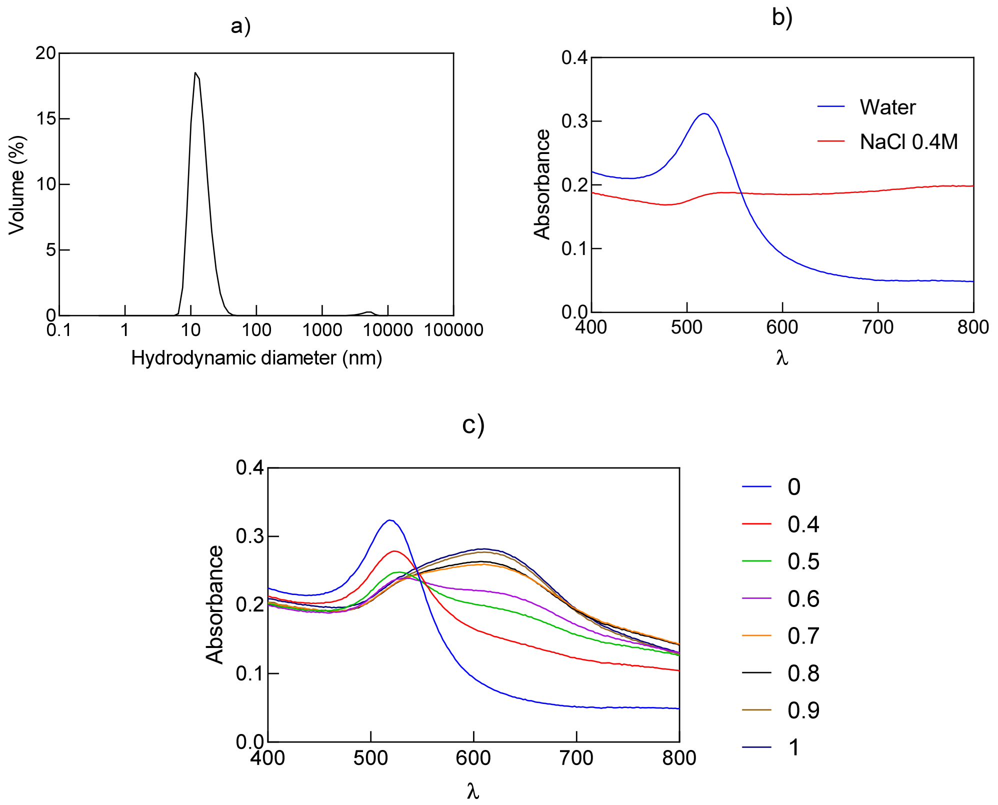


**Figure S3.** (a) Particle size distribution of AuNPs in Stock 1, (b) spectrophotometric scan (λ = 400-800 nm) upon addition of water or NaCl 1:1 (v/v), and (c) aggregation profile of aptamer 40 nt-functionalized AuNPs (117:1 molar ratio) at different NaCl concentrations (0-1M).

**Table S1.** ANOVA for the incubation of FB1 with Aptamer 40 nt in three buffers.

| Sample | | One-Way ANOVA (*p*) | | |
|---|---|---|---|---|
| | | **Tris** | **PBS** | **Mix** |
| Tris | T0 | | vsP0 (<0.05) | vsM0 (<0.05) |
| | T10 | | vsP10 (<0.05) | vsM10 (<0.05) |
| | T100 | | vsP100(<0.05) | vsM100(<0.05) |
| PBS | P0 | vsT0 (<0.05) | | vsM0 (<0.05) |
| | P10 | vsT10 (<0.05) | | vsM10 (<0.05) |
| | P100 | vsT100(<0.05) | | vsM100(<0.05) |
| Mix | M0 | vsT0 (<0.05) | vsP0 (<0.05) | |
| | M10 | vsT10 (<0.05) | vsP10 (<0.05) | |
| | M100 | vsT100 (**0.33**) | vsP100(<0.05) | |

Incubation: Aptamer 40 nt: AuNP molar ratio (117:1), FB1-aptamer incubation (60 min, 37 °C), AuNP incubation (120 min, 37 °C). Tris-HCl buffer: 31.1 mM, PBS: 12.79 mM, Mix: Tris-HCl buffer 31.1 mM + PBS 12.79 mM (NaCl yield).

a)

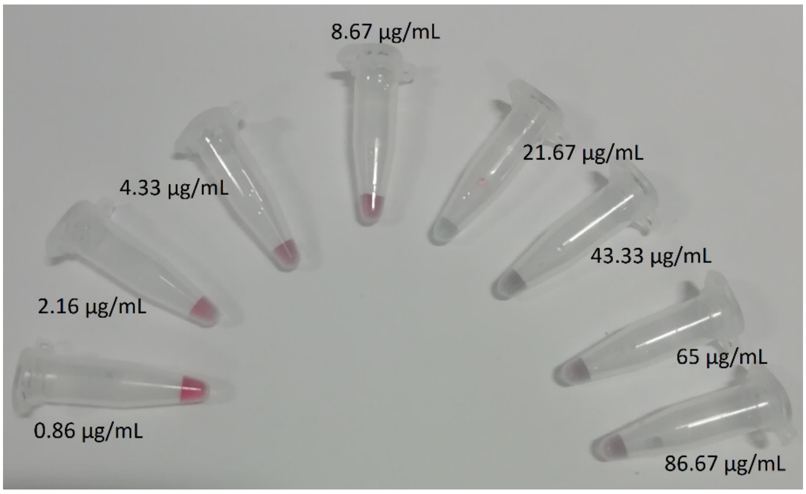


b)

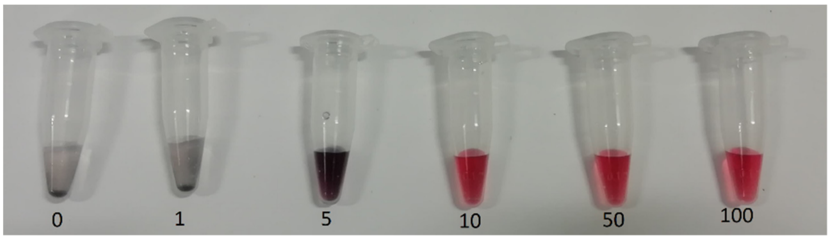


**Figure S4.** (a) Colorimetric effect from the incubation of aptamer 40 nt and FB1 (0.86-86.67 µg/mL, 60 min, 37 °C) with Stock 1 (117:1 Aptamer:AuNP molar ratio, 120 min, 37 °C) after the addition of NaCl (0.4 M, 1:1 v:v) and (b) the incubation of FB1(0-100 µg/mL) with Stock 1 (117:1 aptamer:AuNP molar ratio, 120 min, 37 °C) Note: FB1 was dissolved in a mixture of Tris-HCl (31.1 mM) and PBS (NaCl 12.79 mM yield) buffers.

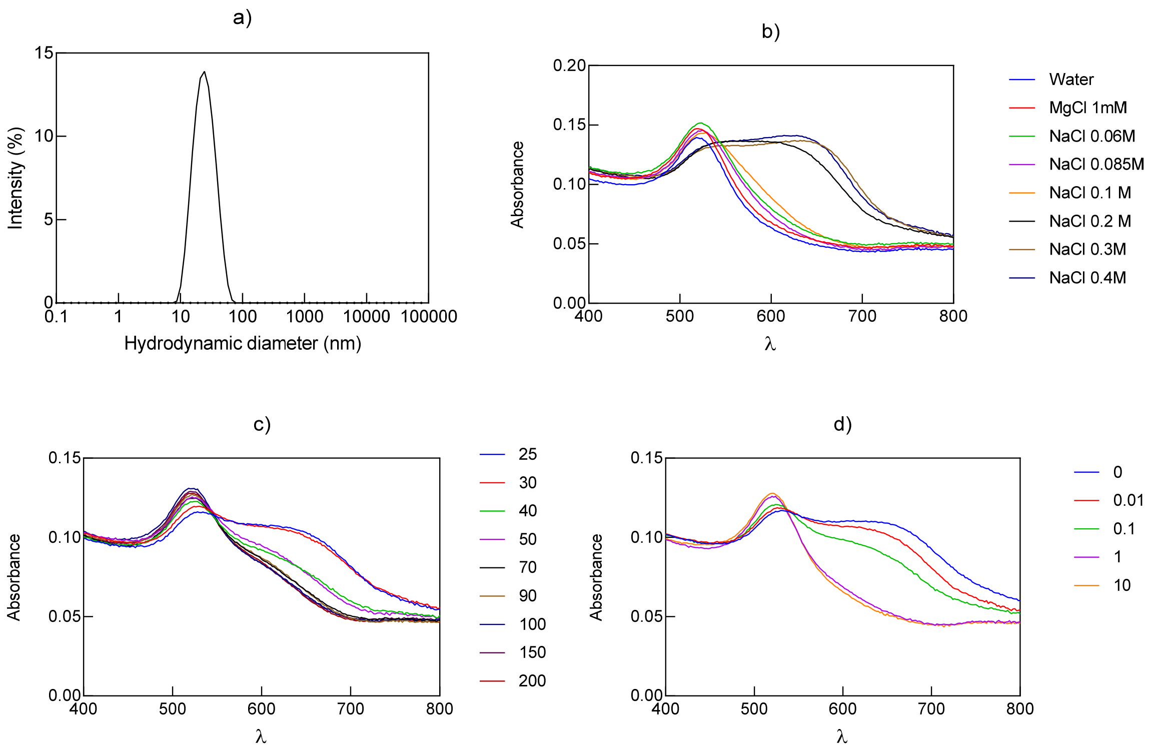


**Figure S5. (**a) Particle size distribution of AuNP in Stock 2, (b) Wavelength (λ = 400-800 nm) scan of AuNP upon addition of water, $MgCl_2$ and NaCl (1:1 v/v) , (c) Wavelength (λ = 400-800 nm) scan of AuNP functionalized with different molar ratios of aptamer 96 nt after the addition of NaCl (0.2 M), (d) aggregation profile of functionalized AuNP with aptamer 96 nt (30:1 molar ratio) and different concentrations of FB1 after the addition of NaCl 0.2 M.

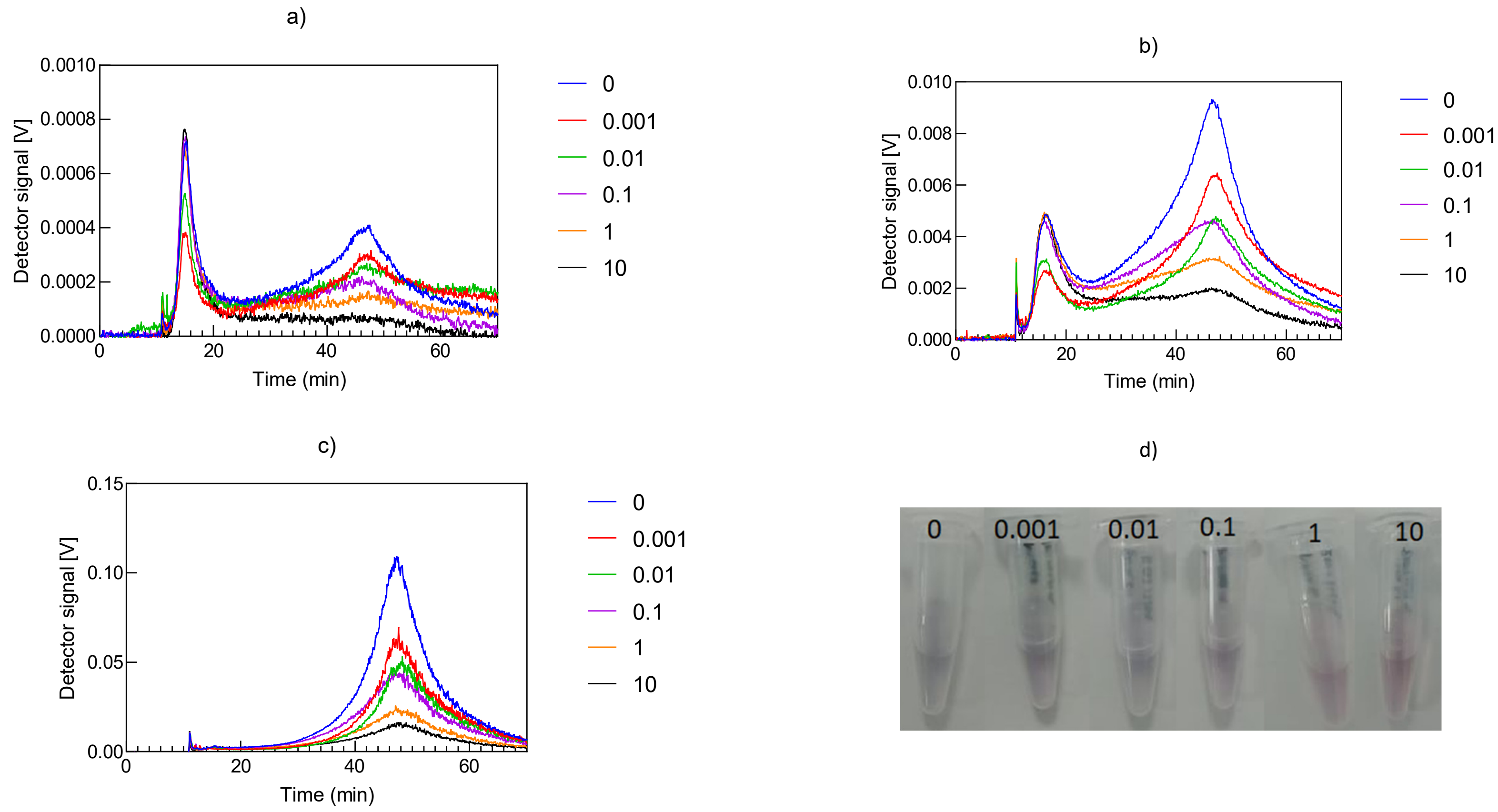


**Figure S6.** Fractograms of the FB1-Aptamer 96 nt-AuNP conjugates at different FB1 concentrations (0-10 µg/mL) after the addition of NaCl 0.2 M detected by AF4 through UV/VIS, λ =520 nm (a), λ = 600 nm (b), MALS 28° (c) signals, and (d) their colorimetric aggregation profile.

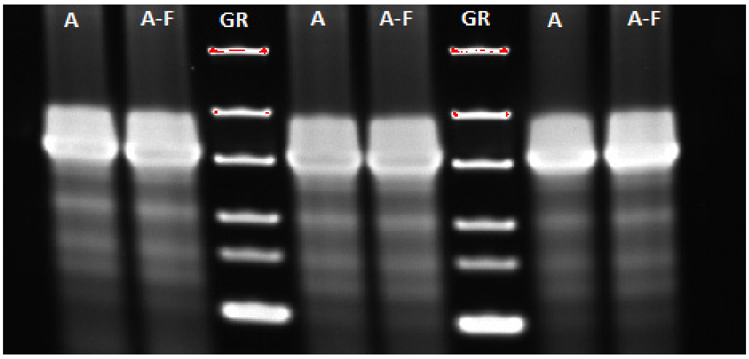


**Figure S7.** Characterization of aptamer 96 nt (A) and aptamer 96 nt-FB1 (A-F) in 14% polyacrylamide gel revealed in ChemiDocTm (Bio Rad) and analyzed in ImageJ. GR: Gene ruler ultra low range DNA Ladder, ready-to-use (SM1213, Thermofisher); Total volume per well 6 µL: 5 µL of aptamer 96 nt (9.3874 µM) or its combination with FB1 (340.11 µM) in $MgCl_2$ 1 mM + 1 µL DNA loading dye. FB1/Aptamer 96 nt molar ratio=36.2305 (equivalent to incubating with 10.02 µg/mL). Electrophoresis at 120 V for 3 h 30 min in TAE buffer, followed by 1 h fixation (10 % acetic acid,40% methanol, 50% water), and 1 h in SYBR gold 1X.

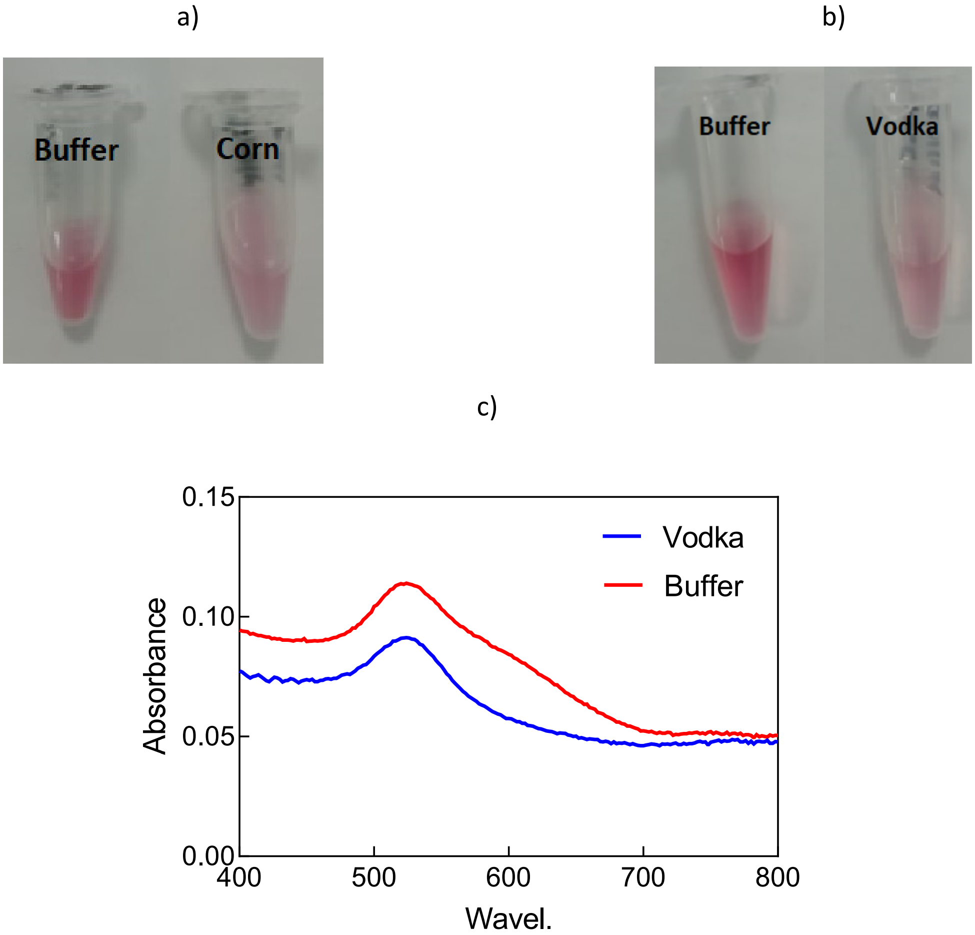


**Figure S8.** Aptamer 96 nt-FB1-AuNP conjugates for the incubation with FB 1 a) 10 µg/mL in buffer and corn extracted with 5% methanol and b) 1 µg/mL in buffer and vodka. (NB: Aptamer 96 nt: AuNP molar ratio 30:1, Aptamer-FB1 incubation: 37 °C for 30 min, Incubation with Stock 2: 1 h at R. Binding buffer: $MgCl_2$ 1 mM).

## References


1 Wu, S., Duan, N., Ma, X., Xia, Y., Wang, H., Wang, Z. and Zhang, Q. 2012. Multiplexed fluorescence resonance energy transfer aptasensor between upconversion nanoparticles and graphene oxide for the simultaneous determination of mycotoxins. Analytical Chemistry. 84(14), pp.6263-6270.

2 Wu, S., Duan, N., Li X., Tan, G., Ma, X., Xia, Y., Wang, Z. and Wang, H. 2013. Homogenous detection of fumonisin B1 with a molecular beacon based on fluorescence energy transfer between NaYF4:YB, Ho upconversion nanoparticles and gold nanoparticles. Talanta. 116(15), pp.611-618.

3 Wang, W., Wu, S., Ma, X., Xia, Y., Wang, Z. 2013. Novel methods for fumonisin B1 detection based on AuNPs labelling and aptamer recognition. Journal of Food Science and Biotechnology. 32(5).

4 Yue, S., Jie, X., Wei, L., Bin, C., Dou, W.D., Yi, Y., QingXia, L., JianLin, L. and TieSong, Z. 2014. Simultaneous detection of ochratoxin A and fumonisin B1 in cereal samples using an aptamer-photonic crystal encoded suspension array. Analytical Chemistry. 86(23), pp.11797-11802.

5 Zhao, Y., Luo, Y., Li, T. and Song, Q. 2014. Au NPs driven electrochemiluminescence aptasensors for sensitive detection of fumonisin B1. RSC Advances, 4, pp.57709-57714.

6 Chen, X., Huang, Y., Ma, X., Jia, F., Guo, X. and Wang, Z. 2015.Impedimetric aptamer-based determination of the mold toxin fumonisin B1. Microchimica Acta. 182(9-10), pp.1709-1714.

7 Chen, X., Bai, X., Li, H. and Zhang, B. 2015. Aptamer-based microcantilever array biosensor for detection of fumonisin B1. RSC Advances. 5, pp.35448-35452.

8 Shi, Z.-Y., Zheng Y.-T., Zhang, H.-B., He, C.-H., Wu, W.-D. and Zhang, H.-B. 2015. DNA electrochemical aptasensor for detecting fumonisin B1 based on graphene and thionine nanocomposite. Electroanalysis. 27(5), pp.1097-1103.

9 Gui H, Jin Q, Zhang Y, Wang X, Yang Y, Shao C, Cheng C, Wei F, Yang Y, Yang M, Song H (2015) Development of an aptamer/ fluorescence dye PicoGreen-based method for detection of fumonisin B1. Sheng Wu Gong Cheng Xue Bao. 31(9), pp.1393–1400.

10 Ren, C., Li, H., Lu, X., Quian, J., Zhu, M., Chen, W., Liu, Q., Hao, N. Li, H. and Wang, K. 2017. A disposable aptasensing device for label-free detection of fumonisin B1 by integrating PDMS film-based micro-cell and screen-printed carbon electrode. Sensors and actuators B: Chemical. 251, pp.192-199.

11 Yang, Y., Li, W., Shen, P. Liu, R., Li., Y., Xu, J., Zheng, Q., Zhang, Y., Li, J. and Zheng. T. 2017. Aptamer fluorescence signal recovery screening for multiplex mycotoxins in cereal samples based on photonic crystal microsphere suspension array.  Sensors and Actuators B: Chemical. 248, pp.351.358.

12 Wang, C., Qian, J., An, K., Huang, X., Zhao, L., Liu, Q., Hao, N., Wang, K. 2017 Magneto-controlled aptasensor for simultaneous electrochemical detection of dual mycotoxins in maize using metal sulfide quantum dots coated silica as labels. *Biosensors and Bioelectronics.* **89**, pp.802-809.

13 Molinero-Fernández, A., Moreno-Guzmán, M., Ángel López, M., Escarpa, A. 2017. Biosensing strategy for simultaneous and accurate quantitative analysis of mycotoxins in food samples using unmodified graphene micromotors. Analytical Chemistry.89, pp.10850-10857.

14 Tian, H., Sofer, Z., Pumera, M., Bonanni, A. 2017. Investigation on the ability of heteroatom-doped graphen for biorecognition. Nanoscale. 9, pp.3530-3536.

15 王红旗, 王俊艳, 洪慧杰, 尹海燕, Maragos, C., 张 玲, 刘继红. 2017. 伏马毒素 B1 核酸适配体链置换探针的筛选及应用. 农产品质量与安全. 1, pp.44-48.

16 Liu, R., Li, W., Cai, T., Deng, Y., Ding, Z., Liu, Y., Zhu, X., Wang, X., Liu, J., Liang, B., Zheng, T., LI, J. 2018. TiO2 nanolayer-enhanced fluorescence for simultaneous multiplex mycotoxin detection by aptamer microarrays on a porous silicon surface. Applied Materials and Interfaces. 10, pp.14447-14453.

17 Cheng, Z., Bonanni, A. 2018. All-in-One: Electroactive nanocarbon as simultaneous platform and label for single-step biosensing. Nanomaterials. 24, pp.6380-6385.

18 Molinero-Fernández, A., Jodra, A., Moreno-Guzmán, M., López, M.A., Escarpa, A. 2018. Magnetic reduced graphene oxide/nickel/platinum nanoparticles micromotors for mycotoxin analysis. Chemistry A European Journal. 24, pp.7172-7176.

19 Hao, N., Lu, J., Zhou, Z., Hua, R., Wang, K. 2018. A pH-resolved colorimetric biosensor for simultaneous multiple target detection. ACS Sensors. 3, pp.2159-2165.

20 Niazi, S., Khan, I.M., Yan, L., Khan, M.I., Mohsin, A., Duan, N., Wu, S., Wang, Z. 2019. Simultaneous detection of fumonisin B1 and ochratoxin A using dual-color, time-resolved luminescent nanoparticles (NaYF4: Ce, Tb and NH2-Eu/DPA@SiO2) as labels. Analytical and Bioanalytical Chemistry. 411, pp.1453-1465.

21 Wang, C., Huang, X., Tian, X., Zhang, X., Yu, S., Chang, X., Ren, Y., Qian, J. 2019. A multiplexed FRET aptasensor for the simultaneous detection of mycotoxins with magnetically controlled graphene

oxide/Fe3O4 as a single energy acceptor. Analyst, 144(20), 6004-6010. https://doi.org/10.1039/C9AN01593K
22 Wei, M., Zhao, F., Feng, S., Jin, H. 2019. A novel electrochemical aptasensor for fumonisin B 1 determination using DNA and exonuclease-I as signal amplification strategy. BMC chemistry, 13(1), 1-6. https://doi.org/10.1186/s13065-019-0646-z
23 Han, Z., Tang, Z., Jiang, K., Huang, Q., Meng, J., Nie, D., Zhao, Z. 2020. Dual-target electrochemical aptasensor based on co-reduced molybdenum disulfide and Au NPs (rMoS2-Au) for multiplex detection of mycotoxins. Biosensors and Bioelectronics, 150, 111894. https://doi.org/10.1016/j.bios.2019.111894
24 Wei, M., Xin, L., Feng, S., Liu, Y. 2020. Simultaneous electrochemical determination of ochratoxin A and fumonisin B1 with an aptasensor based on the use of a Y-shaped DNA structure on gold nanorods. Microchimica Acta, 187(2), 1-7. https://doi.org/10.1007/s00604-019-4089-y
25 He, D., Wu, Z., Cui, B., Xu, E. 2020. Aptamer and gold nanorod–based fumonisin B1 assay using both fluorometry and SERS. Microchimica Acta, 187(4), 1-8. https://doi.org/10.1007/s00604-020-4192-0
26 He, D., Wu, Z., Cui, B., Xu, E. 2020. Aptamer and gold nanorod–based fumonisin B1 assay using both fluorometry and SERS. Microchimica Acta, 187(4), 1-8. https://doi.org/10.1007/s00604-020-4192-0
27 Tao, Z., Zhou, Y., Li, X., & Wang, Z. (2020). Competitive HRP-Linked Colorimetric Aptasensor for the Detection of Fumonisin B1 in Food based on Dual Biotin-Streptavidin Interaction. Biosensors, 10(4), 31. https://doi.org/10.3390/bios10040031
28 He, D., Wu, Z., Cui, B., Jin, Z., Xu, E. 2020. A fluorometric method for aptamer-based simultaneous determination of two kinds of the fusarium mycotoxins zearalenone and fumonisin B1 making use of gold nanorods and upconversion nanoparticles. Microchimica Acta, 187, 254. https://doi.org/10.1007/s00604-020-04236-4
29 Jiang, D., Huang, C., Shao, L., Wang, X., Jiao, Y., Li, W., Chen, J. and Xu, X., 2020. Magneto-controlled aptasensor for simultaneous detection of ochratoxin A and fumonisin B1 using inductively coupled plasma mass spectrometry with multiple metal nanoparticles as element labels. Analytica Chimica Acta, 1127, pp.182-189.
30 Wu, Y., Yu, J., Li, F., Li, J. and Shen, Z., 2020. A Calibration Curve Implanted Enzyme-Linked Immunosorbent Assay for Simultaneously Quantitative Determination of Multiplex Mycotoxins in Cereal Samples, Soybean and Peanut. *Toxins*, *12*(11), p.718.
31 Zheng, Y.T., Zhao, B.S., Zhang, H.B., Jia, H. and Wu, M., 2020. Colorimetric aptasensor for fumonisin B1 detection by regulating the amount of bubbles in closed bipolar platform. *Journal of Electroanalytical Chemistry*, *877*, p.114584.
